# Tomato Allergy: The Characterization of the Selected Allergens and Antioxidants of Tomato (*Solanum lycopersicum*)—A Review

**DOI:** 10.3390/antiox11040644

**Published:** 2022-03-28

**Authors:** Katarzyna Włodarczyk, Beata Smolińska, Iwona Majak

**Affiliations:** 1 Institute of Natural Products and Cosmetics, Department of Biotechnology and Food Sciences, Lodz University of Technology, ul. Stefanowskiego, 2/22 90-537 Lodz, Poland; beata.smolinska@p.lodz.pl; 2 Institute of Food Technology and Analysis, Department of Biotechnology and Food Sciences, Lodz University of Technology, ul. Stefanowskiego, 2/22 90-537 Lodz, Poland; iwona.majak@p.lodz.pl

**Keywords:** tomato allergens, profilin, β-fructofuranosidase, lipid transfer protein, pathogenesis-related proteins, antioxidants

## Abstract

Tomatoes are one of the most broadly produced and consumed crop plants. They are the source of health-promoting nutrients such as antioxidants, including ascorbic acid, polyphenols, or carotenoids. Despite the beneficial role of tomatoes in the daily diet, they have been confirmed as one of the most prevalent allergenic vegetables. Food allergies can cause many clinical symptoms, e.g., in the gastrointestinal tract, skin, and lungs, as well as anaphylactic shock. A huge amount of clinical research has been carried out to improve the understanding of the immunological mechanisms that lead to the lack of tolerance of food antigens, which can result in either immunoglobulin E (IgE)-mediated reactions or non-IgE-mediated reactions. Lifestyle and diet play an important role in triggering food allergies. Allergy to tomatoes is also linked to other allergies, such as grass pollen and latex allergy. Numerous attempts have been made to identify and characterize tomato allergens; however, the data available on the subject are not sufficient.

## 1. Introduction

Tomatoes (*Solanum lycopersicum*) are one of the most broadly cultivated and consumed crop plants. This popular plant can easily adapt to different climatic conditions, which allows it to be cultivated worldwide. Tomatoes can be consumed raw or processed in various forms. The fruits of this particular plant are often used to produce a wide range of processed products (juice, soups, ketchup, sauces, dried tomatoes, etc.). The genus of tomatoes is divided into 13 species and thousands of cultivars which are spread worldwide [1]. They can have different colors and sizes, e.g., the most popular are red tomatoes, but they can also be yellow, green, or even purple. This popular vegetable is rich in nutrients and contains various health-related compounds. The level of certain nutrients depends on the stage of maturity, the type of cultivar, or the environmental conditions in which they are grown [2,3]. Tomatoes are a rich source of antioxidants, such as ascorbic acid, polyphenols, or carotenoids. In addition, tomatoes contain many other nutrients, such as iron (Fe), calcium (Ca) and vitamin A, thiamine (vitamin B), and ascorbic acid (vitamin C) (Table 1). This vegetable is undeniably an important part of our everyday diet. It was calculated that consumption of 230 g of tomato can easily supply nearly 60% of the recommended daily intake of vitamin C in adults and 85% in children. Moreover, consumption of 100 mL of tomato juice constitutes about 20% of the recommended daily intake of vitamin A [4].

The global tomato production rate (fresh and processed) has been constantly growing for over five decades. It started in 1961, when the production was estimated at 27.6 million tons, and finally reached about 171 million tons in 2014. The ratio between fresh and processed tomato production depends on the country of production (Table 2). In the USA, which is one of the biggest tomato producers, almost 96% of grown tomatoes is processed, whereas in India it is only 1% [6]. In 2015, processed tomato production reached about 41 million tons. The USA, China, Italy, Spain, and Turkey, respectively, were the biggest producers, and they represented approximately 85% of the global production of processed tomatoes [6]. 

## 2. Tomato Antioxidants

Tomatoes are the source of many health-promoting nutrients. They have a significant level of carotenoids, which, in a similar way to lycopene, are responsible for the red color which is the most common for them. The content of lycopene in fresh tomato fruit is approximately 12 mg/100 g. Additionally, tomatoes contain other carotenoids, such as β-carotene, phytoene, and lutein. These compounds, according to many studies, reduce the risk of cancer [7,8,9]. Furthermore, the consumption of tomato, and the carotenoids contained therein, is advisable in preventing cardiovascular diseases and reducing the risk of developing other diseases, such as colon, stomach, or rectal cancer [8,9,10]. 

Tomatoes also contain nutrients, such as the previously mentioned vitamins and polyphenols (anthocyanins and phenol acids). Moreover, they contain a small amount of flavonoids (5–10 mg/kg fresh weight) [11], which are found in their peel. This group includes naringenin, chalcone, flavonol rutin, and the quercetin glycoside.

Although, tomatoes can be processed into variety of products, many studies indicate that this does not necessarily exclude the health benefits of their consumption. Numerous studies confirm that processing, such as cooking or baking, may not have a significant influence on the lycopene or β-carotene. Interestingly, such processes can sometimes even increase the content of those antioxidants in the obtained products. Unfortunately, at the same time, many other studies indicate that specific types of treatment may decrease the content of ascorbic acid in tomatoes [2,12,13,14,15,16].

Some studies report that there can be a beneficial correlation between antioxidant occurrence in plants and allergic outcomes. In a study conducted by Yamamoto et al. (2004) [17], the inhibition of histamine release from rat peritoneal mast cells was reported in cells which were previously treated with the tomato extract. Although histamine is known as a mediator in allergic reactions, the inhibition of its release could potentially lead to the mitigation of the allergic symptoms. The anti-allergic activity of this extract was subsequently investigated in an in vivo mouse ear-swelling response. The strongest inhibition was obtained by naringenin chalcone, which is a polyphenol found in tomato skin.

In another more specific study, Hossin et al. (2012) [11] investigated the anti-allergic potential of red tomato peel and flesh extracts and yellow tomato peel extract. The researchers conducted, among other tests, a skin prick test on patients suffering from a nasobronchial allergy and compared them to the results from healthy volunteers. Firstly, the patients were divided into three groups; in each group, they received tablets containing the mentioned extracts for 8 weeks. This study confirmed that the red tomato peel extract (RTPE) has a more effective anti-allergic activity compared to other analyzed extracts. They also performed a skin test for an allergy score, the results of which showed a significantly lower score only in the RTPE group at the end of the trial. The same results were obtained for sneezing, the decreasing tendencies of rhinorrhea, and the nasal obstruction. The significant difference between all the used extracts is the amount of flavonoids or carotenoids. In the peel of red tomato, the level of lycopene is much higher than in the flesh or in the peel of yellow tomatoes. The same trend is visible in the case of lutein or chlorogenic acid. These findings lead to the conclusion that certain antioxidants may have an anti-allergic effect.

Even though the antioxidants from tomatoes may have anti-allergic potential, they cannot counteract the allergy for tomatoes. The antioxidants mentioned above may help with the allergenic reaction to house dust or mites, but both the flesh and the tomato skin are, at the same time, the sources of tomato allergens.

## 3. Tomato Allergy

**Figure 1:**
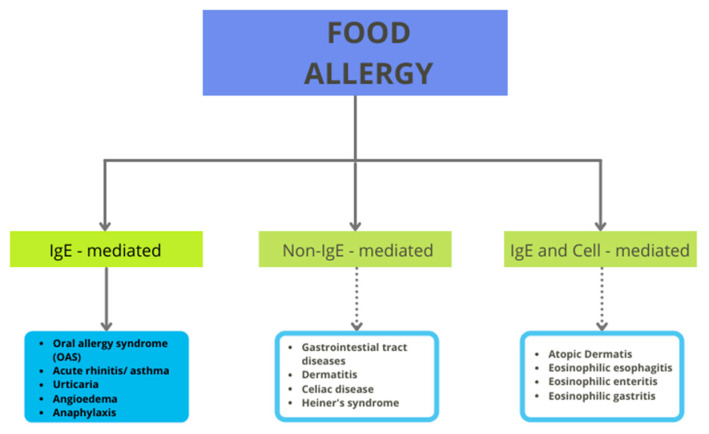
Classification of food allergy.

Despite the health benefits that tomatoes can provide, there is a group of consumers who must avoid tomato in their daily diet. The reason for this necessity is the risk of allergic reactions after consumption. Generally, these reactions can be IgE-mediated, non-IgE-mediated, or both (see Figure 1). IgE-mediated reactions can cause numerous symptoms, such as cutaneus (urticaria and flushing), gastrointestinal (the oral allergy syndrome—OAS, pollen food allergy syndrome, and anaphylaxis), or respiratory reactions. The non-IgE-mediated reactions include, for example, contact dermatitis, food-protein-induced enteropathy, and celiac disease [18]. Tomato allergy is immunoglobulin E (IgE)-mediated and it partly originates from pollen cross-reactivity. 

The studies show that among the European population, tomato allergy ranges from 1.5% in northern Europe [19] up to 16% of the adults in Italy [20]. Different studies indicate that the prevalence of tomato allergy is approximately 1.7% to 9.3% in different populations of Europe with an average of 4.9% [21]. Nevertheless, the tomato has been confirmed as one of the most prevalent allergenic vegetables [22,23]. As this allergy mostly affects the population of southern Europe, the majority of such studies are dedicated to Italy and Spain [24,25]. 

Usually, patients with a tomato allergy are exposed to local symptoms, mainly oral allergy syndrome (OAS), which occurs at the mucosa of the lips, tongue, or pharynx after ingestion, and to cutaneous symptoms (pruritus, erythema) or even hives after contact or ingestion. Sometimes, the symptoms can also cause a reaction of the gastrointestinal tract and the cardiovascular system [26,27,28]. Affected individuals must avoid tomato fruits, which may lead to a decrease in the quality of their lives. Due to the classification of the types of immune responses developed by Coombs and Gell (1963) [29], patients sensitized to tomatoes have the symptoms belonging to allergy type I. This type of immune response is named immediate. What is typical for this kind of reaction is that the symptoms occur a few seconds to a few minutes after the contact with the allergen, and the IgE antibodies are involved in the response. Contrary to the three other types of immune responses classified by Coombs and Gel, this one is caused by allergens, pollens, and, more rarely, drugs. As opposed to the symptoms belonging to other classes of immune responses, the type 1 responses are caused by factors such as drugs, infectious agents, inhaled molds, or contact allergens (nickel in jewelry, black hair dyes, fragrances, preservatives). Moreover, the other pathogenic mechanisms of allergic reaction (type II, III, or IV) are IgE-independent, and they involve T cells, IgG, IgM, or IgA [30].

Most patients show allergy symptoms after consumption of fresh tomato fruits, whereas they can mostly tolerate processed tomato products. Unfortunately, the latter products also contain allergens, for instance lipid transfer protein (LTP)—Sola l 3 [31] and putative vicilin and legumin [32].

To date, 27 potential tomato allergens have been reported in different databases. The International Union of Immunological Societies (IUIS) listed seven confirmed tomato allergens (Table 3). Three of them (Sol l 1, Sol l 2, and Sol l 3) have been characterized more thoroughly than the rest. The list, apart from Profilin (Sol l 1), β-fructofuranosidase (Sol l 2), and Lipid-transfer protein (Sol l 3), also contains Intracellular pathogenesis-related protein (Sol l 4), Cyclophilin (Sol l 5), 7 kDa lipid transfer protein (Sol l 6), and 11 kDa lipid transfer protein (Sol l 7). Many other IgE-binding proteins have been reported in the literature as putative tomato allergens. Some of these proteins are already featured in the Allergome database, but there are only preliminary data available on their allergenicity. 

The first studies which highlighted the problem of tomato allergy were conducted by Bleumink et al. (1966) [33]. They isolated the glycoprotein fraction from tomatoes with an enhanced skin-prick-test activity. One year later, the same scientists reported that those glycoproteins have molecular weights of approximately 20–30 kDa [34]. Over the last few decades, the number of studies on tomato allergens has increased significantly. For instance, Foetisch et al. (2001) [35] carried out a complex study using a combination of different detection methods, such as two-dimensional immunoblotting combined with N-terminal microsequencing and specific IgE measurements (CAP, EAST, ELISA). According to this study, β-fructofuranosidase and other enzymes such as polygalacturonase or pectinesterase were defined as IgE-binding components in tomato extract. The results of the mentioned research were also confirmed in the study provided by Kondo et al. (2001) [36]. Moreover, the research of Foetisch et al. (2001) [35] proved that profilin is an important allergen in tomatoes. This observation was supported by conducting the inhibition of IgE-binding profilin from recombinant birch pollen. Profilin was the first protein confirmed and described as a tomato allergen, becoming known as Sola l 1 (previously Lyc e 1).

The next protein, which was officially named as Sola l 2 (previously Lyc e 2), was β-fructofuranosidase. In addition to the previously mentioned study of Foetisch et al. (2001) [35], the research of Westphal et al. (2003) [37] completed the data about β-fructofuranosidase.

The third discovered allergen in tomatoes was non-specific lipid transfer protein (nsLTP). The evidence for nsLTP being a tomato allergen was provided by Asero et al. (2000) [38], who conducted the research using the screening of numerous plants for their reactivity with IgE antibodies against the LTP from Rosaceae. Foetisch et al. (2001) [35] confirmed these results using the enzyme allergosorbent (EAST) inhibition assay. Furthermore, this research indicates that the LTP extracted from cherry had the ability to inhibit IgE binding in tomato extract.

Numerous different studies have shown that many tomato proteins are likely to be involved in causing tomato allergy. Nevertheless, only three tomato allergens have been more specifically characterized. Examination showed that about 22% of tomato-allergic patients are sensitized to Sola l 1 (profilin) [39]; approximately 17% of the mentioned patients are sensitized to Sola l 2 (β-fructofuranosidase) [39,40]; and up to 15% of Italian tomato-allergic patients were mono-sensitized to the non-specific lipid transfer protein from tomato (Sola l 3) [31,41]. Still, pectinesterase, polygalacturonase 2A, superoxidedismutase [36,39], an osmotin-like protein, glucanase, and chitinase, as well as a 9 kDa heat-labile and pepsin-resistant protein, are being discussed as potential tomato allergens [40,41,42,43]. 

## 4. Profilins—Sola l 1

Profilins are a family of ubiquitous cytosolic actin-binding proteins (14- to 17 kDa) which are expressed in eukaryotes and certain viruses [44]. In 1977, Carllson et al. (1977) [45] proved that profilins are involved in promoting the polymerization of actin filaments and monomers. They catalyze the process of ADP/ATP exchange in actin and consequently increase the rate of polymerization. This ability makes them involved in the process of building of the cytoskeleton. Their involvement in such an essential process explains their ubiquitous expression and high levels of conservation [46]. This capability indicates the importance of profilins in the process of cell proliferation, growth, motility, and cytokinesis.

**Figure 2:**
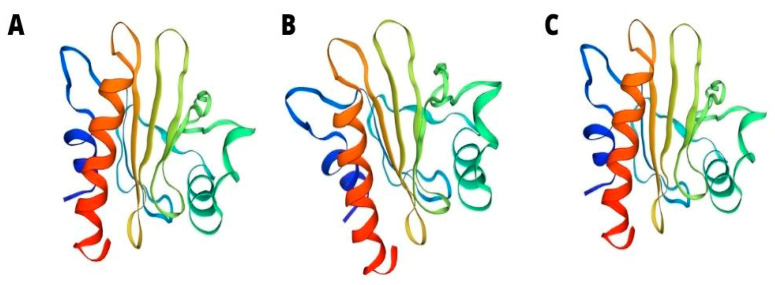
3D structure of tomato Sola l 1 (**A**) Latex Hev b 8 (**B**) and apple Mal d 4 (**C**).

Most profilins consist of seven beta sheets and four helices, and their isoforms consist of 100–130 amino acids. Studies conducted on profilin isoforms, which are derived from different organisms, prove that despite the undeniable difference in sequences of amino acids, their three-dimensional (3D) structures seem to be similar (Figure 2). 

In 1996, Darnowski et al. (1996) [47] analyzed the distribution of profilin in the tissues of tomato. In the same decade, other scientific groups analyzed the structure of free and linked N-glycans in tomatoes [48,49]. They showed that analyzed structures contain the plant-characteristic glycan core with xylose and fucose, which is known as an IgE-binding epitope. The results of the mentioned study clearly indicated the presence of allergens in tomato tissue. 

As was mentioned, profilins are highly conserved proteins, and they have been identified as allergens of many grasses (*Lolium perenne*, *Phleum pratense*), weeds (*Artemisia vulgaris*), fruits (apples, peach, sweet cherry, and banana), natural latex (*Hevea brasiliensis*) [45,50], and vegetables (bell pepper, celery, carrot, and soybeans) [51] (Table 4).

The first time profilin was recognized as an allergen derived from birch pollen (Bet v 1) was in 1991, by Valenta et al. (1991) [52]. Since then, many other proteins belonging to the profilin family have been identified as allergens as well. For tomato, strong evidence for profilin being a significant allergen was presented by Foetisch et al. (2001) [35]. Using IgE immunoblotting they detected the presence of IgE specific to profilin in serum from 44% of the examined patients. To confirm that the selected protein belonged to the profilins, immunodetection tests were carried out. They achieved the confirmation by conducting immunodetection with rabbit anti-celery or rabbit anti-ragweed profilin. Consequently, the authors conducted the inhibition of the IgE-binding to tomato extract with recombinant birch pollen profilin (Bet v 2) and celery profilin (Api g 4). Officially, profilin was recognized as a tomato allergen just a few years later [53]. Two independent scientific groups cloned tomato profilin and expressed it in *E. coli* [44,45]. The obtained proteins were purified and examined by IgE-binding and inhibition capacity with sera from allergic patients and by using a basophil histamine release assay.

It has been proved in many studies that tomato allergy is related to allergies to grass pollen [54] and latex [55]. This phenomenon occurs because of profilin, which was identified as a relevant allergen in a group of patients allergic to birch pollen with adverse reactions to tomatoes [35]. 

Profilins are recognized by the IgE antibodies of approximately 20% of patients with birch pollen and plant-derived allergies. They are called panallergens [50], because they are responsible for cross-sensitization between plant pollen and vegetable food extracts [56]. The identification of homologous structures of profilin from different vegetables (potato, tomato, bell pepper) has been proved numerous times in IgE immunoblot inhibition studies. Some isoforms of tomato profilin are pollen specific, whereas other isoforms of profilin are expressed constitutively in all tissues of the plant [56]. 

Tomato profilin Sola l 1 resists heat and digestion by the action of the papain enzyme, which was discovered by Kiyota et al. (2017) [51]. The results of this research showed that the reactivity of Sola l 1 in heated tomato extracts was stable and independent of temperature or time. The epitope of the anti-Sola l 1 antibody was also stable in the presence of papain. The results of this experiment suggest that patients with tomato allergy can experience allergy symptoms regardless of whether they eat fresh tomatoes or processed ones.

There have been many studies which have proven that profilin in tomatoes is a major allergen [24,27,35]. Yu et al. (2014) [57] cloned the profilin from tomato pollen and compared its sequences with the amino acid sequences of fruit profilin. Interestingly, the identity between these two sequences was only 78%. A Blast search in GenBank revealed another sequence from tomato fruit profilin (Acc. no. AJ417553), but this time both sequences showed 89% identity. That was the proof of the existence of different profilin isoforms in tomato fruit. At the same time, the sequence of bell pepper profilin Cap a 1 (Acc no. AJ417552) was published by the same group, and the sequence identity between the tomato profilin and that from bell pepper was 94%. Surprisingly, the result was much higher than for the two sequences derived from tomato. This may be explained by the fact that both, tomato and bell pepper, are in a tight taxonomic vicinity [58]. Profilins were considered to be partly responsible for the IgE cross-reactions between tomato fruit or bell pepper and pollen allergens [21,47].

As it was described above, profilin is a well-known allergen, and many studies on its nature have been carried out. Nevertheless, this particular protein causes many problems with its correct identification in allergic patients. Due to its cross-reactivity, the available immunology-based methods of allergen identification often fail, for example by giving false-positive results. Despite all the accessible data in the field of identification methods, there is still a lot to improve.

## 5. β-Fructofuranosidase—Sola l 2

β-fructofuranosidase (EC 3.2.1.26), known as Sola l 2, is a second protein which was officially recognized as a tomato allergen. To the best of the authors’ knowledge, the number of studies concerning this protein in terms of its allergenic potential is limited. However, β-fructofuranosidase is a well-known enzyme which catalyzes the reaction of sucrose hydrolyzation, obtaining glucose and fructose as a result. This particular protein is also known as an acid invertase, and it can be found in many different plants [59]. Depending on the plant they are present in and on their subcellular localization, they can possess different properties, such as optimal pH or an isoelectric point. 

**Figure 3:**
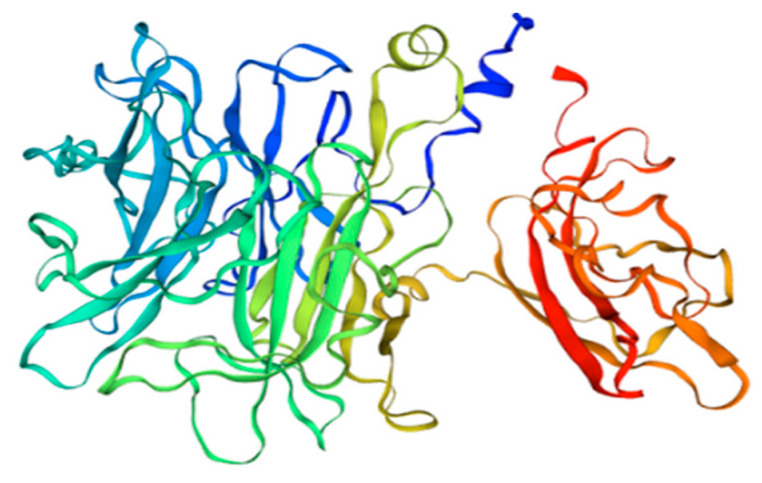
3D model of tomato Sola l 2 structure. Homology Model based on nsLPT derived from Pachysandra terminali, Seq Identity 64.02%.

β-fructofuranosidase is also present in tomatoes, where it is involved in the process of hexose accumulation at the stage of fruit ripening [60]. In tomatoes, Sola l 2 can be found in two isoforms and with different C-terminus regions (see Figure 3). One isoform is 70 kDa (UniProt Q8rVW4), and it is built of 692 amino acids, while the second one is only 61 kDa (UniProt Q547Q0), due to an 86 bp insertion with stop codon, which reduces its length to 553 amino acids. 

The first evidence of recognizing β-fructofuranosidase as a potential allergen was shown in the research of Petersen et al. (1996) [19]. In this research, the allergenicity of Sola l 2 was tested by checking the IgE reactivity to tomato fruits. To identify the components which were binding with the IgE antibodies, the researchers used two-dimensional PAGE immunoblotting. They detected a 52-kDa allergen, which was further analyzed using microsequencing. This research confirmed the presence of β-fructofuranosidase in tomatoes and noted the allergenic potential of this protein. A few years later, the allergenicity of β-fructofuranosidase was further confirmed by Foetisch et al. (2001) [35]. 

A complex study on Sola l 2 was conducted by Westphal et al. (2003) [37]. Both isoforms of β-fructofuranosidase were examined, starting from cloning them into bacterial plasmid and followed by the purification of the expressed recombinant proteins by using electroelution. The IgE reactivity in the sera of the patients who had adverse reactions to tomato were investigated for both recombinant and natural protein. Patients with tomato allergy were divided into two groups, and in both, over 30% of the patients’ antibodies reacted with proteins, presumably glycoproteins, whose mass was over 20 kDa. In the same study, the results showed that both isoforms of Sol l 2 contained four putative N-glycosylation sites. That assumption was concluded based on the observation of IgE binding to natural Sola l 2, which was inhibited by the pineapple stem bromelain glycopeptide MUXF (Mana1–6(Xylb1–2)Manb1–4GlcNAcb1–4(Fuca1–3) GlcNAc). Further analysis showed that non-glycosylated recombinant protein isoforms, in contrast to natural protein, did not bind the IgE of tomato-allergic patients. Moreover, the results obtained from MALDI-TOF mass spectrometry revealed that the predominant glycan structure of natural β-fructofuranosidase is MMXF (Mana1–6 (Mana1–3)(Xylb1–2)Manb1–4GlcNAcb1–4(Fuca1–3) GlcNAc). The further glycan analysis revealed that MMXF is the most prevalent one, constituting about 84% of all the sugar structures (Table 5).

Westphal et al. (2003) [37] also reported that β-fructofuranosidase (Sol l 2) is able to trigger human basophils to release histamine and that this process is mediated by the glycan structure of the Sol l 2. The conclusion was achieved by comparing the protein with its non-glycosylated recombinant isoform. The latter did not cause mediator release in the tested human basophils.

β-fructofuranosidase was also detected in the carrot cell wall; however, it has not been described as an allergen. There are structural differences between the β-fructofuranosidase found in tomato and the one from carrot. The tomato’s Sola l 2 has four glycosylation sites, while the enzyme from carrot contains only three sites, and only one of these sites was identified as the same structure found in the tomato enzyme MMXF [61].

β-fructofuranosidase is a well-known enzyme, broadly used in several industries, even though the studies on its allergic potential are limited. More research should be carried out to better understand all the allergenic mechanisms related to Sola l 2. 

## 6. Lipid Transfer Protein—Sola l 3

**Figure 4:**
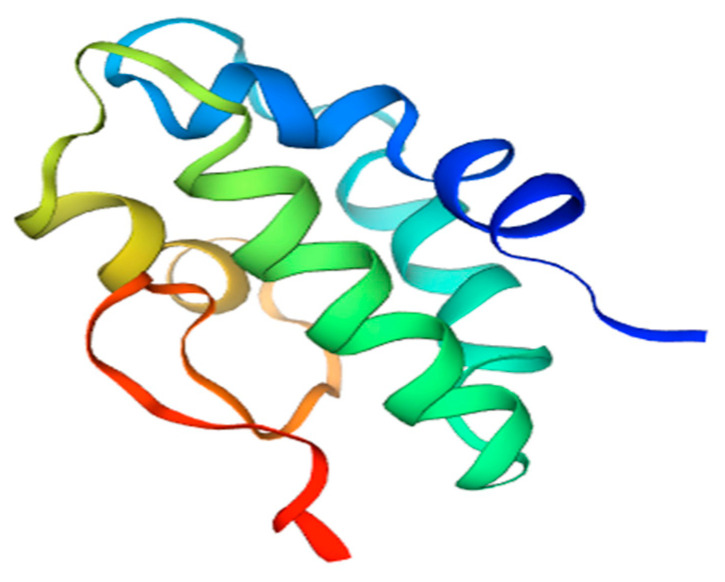
3D model of tomato Sola l 3 structure. Homology Model based on nsLPT derived from Nicotiana tabacum, Seq Identity 71.43%.

Non-specific lipid transfer protein (nsLTP) is a plant food allergen present in variety of fruits and vegetables, such as peach, apple, tomato, carrot, barley, corn, wheat, rice, sorghum, broccoli, onion, grapevine, and others [38]. The widespread occurrence of this protein leads to the situation in which LTP-sensitized patients may experience adverse reactions upon the ingestion of a large array of plant foods (Table 6) [62]. The members of this protein family are located extracellularly; they are mostly associated with plant cell walls and possess a broad lipid-binding specificity closely related to their three-dimensional structure. nsLTPs have a molecular mass of 7–10 kDa, and their structure consists of a domain composed of four α-helices, which are held by a network of four conserved disulphide bridges (see Figure 4). This fold presents a large internal tunnel-like cavity, which can accommodate different types of lipids. 

nsLTPs can present a moderate to high degree of sequence homology (35–95%) [63,64] between each other. This protein has a high resistance to thermal and proteolytic treatments, which makes them extremely stable molecules. There are two main types of plant nsLTPs described: nsLTP1 of 9 kDa and nsLTP2 of 7 kDa [65,66]. What is more, a large number of related nsLTP-like proteins have also been reported [67]. Both nsLTP families share the same general molecular structure but show rather low sequence similarity (about 30% identity). Moreover, they differ in cysteine residues sited along the molecules [68]. The small molecules which belong to the nsLTP1 family contain 91–95 amino acids (lack of Trp), and they have a high isoelectric point (pI 8.5–10). To the nsLTP2 family belongs a limited number of representatives; they are present mostly in cereals and also in tomatoes. However, this method of classification was recently recognized by many researchers as being not adequate due to the discovery of novel nsLTPs which do not fit into any of the mentioned groups. Consequently, this leads to the introduction of new group described as type III [69]. Boutrot et al. (2005) [65] developed a new classification based on the sequence similarity and the spacing between the cysteine residues in the ECM. This system divides nsLTPs into nine types (type I–IX). Additionally, further analysis led to the introduction of two new nsLTPs types to this classification, named X, which were reported only in Solanaceae [70] and XI [71,72]. As the classification developed by Boutrot [65] excluded non-flowering plants, another system of nsLTP classification was introduced by Edstam et al. (2011) [73]. This system is based on a few aspects, such as the glycosylphosphatidylinositol (GPI) modification site, the intron position, and the spacing between the cysteine residues. 

A great number of studies have shown that nsLTPs take part in a large number of biological processes, such as plant growth and development, seed maturation and germination, fruit ripening, cuticle formation, suberin biosynthesis, pollen development, pollen tube adhesion, and growth, but also responses to biotic and abiotic stresses and defense signaling [70,71,74,75,76,77]. nsLPTs have been reported to act as plant defense proteins against infection caused by fungi, bacteria, and viral pathogens, but their mechanism of action is not fully understood [38]. They are classified as pathogenesis-related (PR) proteins and included in the PR-14 family [74]. These proteins all have low molecular masses; they are very stable at low pH, and they are resistant to proteolysis [62]. Due to their defensive role, the majority of nsLTPs are located in the superficial layers in a number of fruits, including Rosaceae (peach, apple, pear, and plum), melon, and watermelon. However, in some fruits, nsLTPs seem to be present in higher amounts in seeds that are eaten along with the pulp. Fruits such as kiwi and tomato are two examples of such a case [75].

LPTs are responsible for severe allergic reactions, including anaphylaxis [26]. They have been identified as important plant-food allergens in fruits, vegetables, nuts, and cereals [76,77]. nsLTPs are considered as one of the major human allergens. These proteins were recognized as the most frequent cause of primary food allergies for adults living in the Mediterranean area, where these proteins are the main allergen responsible for the food-dependent anaphylactic reactions [75,78]. 

Three of the seven officially recognized tomato allergens (registered in the “allergen.org” database) are nsLTPs. These proteins are Sola l 3 (Uniprot P93224), Sola l 6 (Uniprot P93224), and Sola l 7 (Uniprot P93224). The allergen Sola l 3 was identified in the flesh and epicarp of tomato fruits, while the other two were found in seeds [78,79].

Sola l 3 was recognized as a tomato allergen by Asero et al. (2000) [38] by the procedure of the screening of several plant-derived food samples for the cross-reactivity of Rosaceae LTPs with IgE antibodies. Later, Foetisch et al. (2001) [35] proved that nsLTPs are the allergens in tomatoes by using EAST inhibition assays. 

Non-specific lipid transfer protein type 2, later named Sola l 6, was isolated and described by Giangrieco et al. (2015) [80]. In this study, 7k-LTP was isolated from tomato seeds and purified by chromatographic separations. The obtained results indicate that this novel allergen consists of 68 amino acids and a molecular mass of approximately 70 kDa. It also presents a 41% sequence identity with Pru p 3 (nsLTP from peach).

Shortly after the article of Giangrieco et al. (2015) [80], both Sola l 6 and the newly described allergen Sola l 7 were identified as tomato allergens by Martin-Pedraza et al. (2016) [79]. In this research, it was proved that the amino acid identity degree between both the newly identified nsLTP and Sola l 3 reached only 22% for Sola l 6 and 38% for Sola l 7. Nevertheless, both allergens proved to have the ability to bind the IgE from 71.4% of the anaphylaxis patients examined in this study. Moreover, the mix of purified nsLTP proteins induced a positive BAT (Basophil Activation Test). Martin-Pedraza et al. (2016) [79] divided patients into groups based on the different clinical symptoms they displayed (anaphylaxis, uriticaria, and OAS). The obtained results showed that patients with severe, moderate, and mild symptoms present high basophil reactivity with tomato extract. Interestingly, for patients with anaphylaxis, a higher level of basophil activation was induced both by the extract and the purified mixture of nsLTP. The group of patients with anaphylaxis showed sensitivity to nsLTP from other vegetables and fruits, such as peach (Pru p 3) and peanuts (Ara h 9), and lower for sunflower seeds (Hel a 3). The researchers also performed an in vitro IgE-inhibition immunoblotting experiment. The sera used in this examination were from patients showing anaphylaxis symptoms. As inhibitors, the extracts from other parts of tomatoes (such as the pulp, peel, and placenta) and from other foods (e.g., peanut, kiwi, apple, peach, etc.) were used. IgE binding was inhibited significantly by extracts from different parts of tomatoes but also by extracts from peanuts, peaches, and apples, which indicates a high cross-reactivity between those fruits and nuts. Other extracts (kiwi, mustard, and sunflower seeds) showed partial or non-inhibition.

Recently, D’Agostino et al. (2019) [72] identified 64 sequences in the tomato genome which are putative in coding nsLTP. To identify the nsLTPs, the hidden Markov model profiles were used, based on the available RNA-seq expression profile data. The Markov model profiles of PF14368 and PF00234 were searched against the tomato protein complement (iTAG v.2.4). Firstly, the authors identified over one hundred putative nsLTP genes, but, following the further analysis, they removed some of them, and finally, only 64 genes were identified as putative nsLTPs genes. Selected proteins were analyzed to confirm the presence of the ECM determining the spacing of Cys residues. The average length of the obtained proteins ranged from 93 amino acids to 138 aa. The average length of the mature nsLTPs was 91 ± 13, with a molecular mass ranging from 6038 to 9922.

Even though during last two decades a lot of studies have focused on the identification and characterization of non-specific Lipid Transfer Proteins, the data on the subject are still incomplete. The recent work of D’Agostino et al. (2019) [72] showed how little we knew about nsLTPs derived from tomatoes. Due to this research, we came from three well-documented allergens to a possible several dozen more. Despite the allergenic potential of nsLTP, we can be sure that these proteins perform an initial role in plant development.

## 7. The Pathogenesis-Related Proteins—Sola l 4

The data on the Sola l 4 are strongly limited compared to other officially recognized tomato allergens. To date, little research has been conducted to characterize or somehow describe this protein derived from tomato. Although the data on Sola l 4 are not sufficient, there is a large amount of information about other proteins belonging to the same family as Sola l 4. The pathogenesis-related proteins (PR) are well-known plant-derived allergens. In plants, these proteins are involved in plant defense for stress conditions such as insect infections, wounding, microbial infections, the presence of harsh chemicals (herbicides and fungicides), or for other harmful environmental changes (dryness and UV light) [81]. Unfortunately, they are not only known as major food allergens, but also as latex or pollen allergens. The examples of pathogenesis-related proteins are, for instance, the major birch pollen allergen Bet v 1, as well as Mal d 1 (apple), Pru p 1 (peach), Pru av 1 (cherry), and, finally, Sola l 4 from tomatoes. Additionally, all the mentioned proteins belong to the PR-10 family. Characteristic for members of this protein family is the low molecular weight (around 15–18 kDa), their acidic character, and the fact that they are resistant to proteases [81]. PR-10s are very often responsible for the phenomenon of cross-reactivity among allergens from diverse plants. Although those proteins share approximately 50% of the amino acid sequence identity, it is not the direct cause of occurrence of cross-reactivity between them. The main reason for cross-reactivity among members of the PR-10 family is the high three-dimensional structure similarity [81]. The first extensive research where PR-10 was officially recognized as a tomato allergen was conducted by Wangorsch et al. (2015) [82]. In this research, the two isoforms of Sola l 4 (Sola l 4.01; UniProt K4CWC6) and Sola l 4.02 (UniProt K4CWC4) were identified in tomato extracts using cDNA-cloning. It was calculated that Sola l 4.01 was 18 kDa with a pI equal to 5.35, and for Sola l 4.02, those parameters are 17.5 kDa for mass and pI 5.44. Additionally, Wangorsch et al. (2015) [82] examined birch-allergic patients with an allergy or a tolerance to tomato with an ImmunoCAPTM analysis to determine the level of IgE specific to both Sola l 4 allergens and Bet v 1. The results of the ImmunoCAPTM analysis revealed a prevalence of IgE specific to Sola l 4 in sera from 76% birch/tomato-allergic patients. For Bet v 1, the analysis showed the presence of specific IgE in 81% of the patients’ sera. Moreover, the results obtained in this research showed that the majority of patients who were sensitized to Bet v 1 also reacted to Sola l 4, which may again point out the common problem of cross-reactivity.

Another study related to the topic of PR-10 in tomatoes (Sola l 4) was provided by Kurze et al. (2018) [83]. The authors of this research investigated the correlation between certain parameters of tomatoes (such as size, shape, and color) and the allergen level. Additionally, the influence of the cultivation method and the processing techniques on the amount of Sola l 4 in tomato fruits was also taken under consideration. The obtained results clearly suggest that the growing conditions have a minor effect on the Sola l 4 content, whereas the level of this allergen varied significantly between different cultivars. For instance, among 23 examined tomato cultivars the lowest level of Sola l 2 was found in two cultivars, Rugantino (0.24 μg Sola l 4/g FW) and Rhianna with 0.29 μg Sola l 4/g FW; in parallel with the cultivars Farbini (1.71 μg Sola l 4/g FW) and Bambello (1.5 μg Sola l 4/g FW), the concentration was significantly higher. Another observation provided by Kurze et al. [83]. was the verification of thermal instability of Sola l 4, which was reveled in process of drying tomatoes in the oven and in the sun.

Although PR-10s are the major plant-derived allergens and the data related to them are abundant, the information about Sola l 4 is strongly limited. Considering the fact that PR-10s are involved in allergen cross-reactivity, more research is needed.

## 8. Cross-Reactivity

There is one significant detail that should be considered during every study on allergens—the phenomenon of cross-reactivity. In the case of plant allergens, it starts when the similarity of the amino acid sequences reaches a high percentage (as with many profilins) or when the allergen has the asparagine-linked carbohydrate moieties of glycoproteins. They are the base of the cross-reactive carbohydrate determinants (CCDs) structure. The two main motifs of CCDs are the xylose and the core-3-linked fucose, which form the essential part of two independent epitopes [84]. The research of Fredrich Altmann (2007) [85] found that at least 20% of allergic patients generate specific anti-glycan IgE, which is often accompanied by IgG. 

Antibody-binding glycoproteins are widespread in pollens, foods, and insect venoms. Usually, allergen-specific IgE detection assays are used to detect and identify the specific antigen which is causing the allergic reaction. CCDs are the cause of incorrect results in diagnostic tests based on in vitro diagnostics. The above-mentioned specific CCD-IgE antibodies can cause false-positive results in commonly used commercial tests. For example, a patient who is allergic to pollen has in his serum IgE specific for carbohydrate determinants that can potentially react with some other allergens, even if the patient has not been in contact with them. However, there are cases in which patients show no symptoms despite the presence of allergen-specific IgE antibodies. Still, this phenomenon can cause confusion during allergen detection in food. However, there are multiple solutions to deal with this problem. In the early 1980s, the basophil activation test (BAT) was invented and was used as a supporting test in allergen diagnosis. This test is based on the ability of basophils to express specific markers (e.g., CD63). Thanks to the addition of flow-cytometry, the BAT assay became even more precise. It measures IgE function such as the capability to induce the basophil activation in the presence of allergen. During the next few decades other assays were invented, such as one of the most popular, the immunoCAP test. Many variations of this assay have appeared, but still their main aim was to detect specific anti-CCD IgE antibodies in examined sera. The immunoCAP test uses the MUXF3 determinants, which are obtained from the digestion with the popular enzyme, bromelain. The carbohydrate chain of MUXF3 can be found in many other plant proteins; nevertheless, allergy to bromelain is very rare in the population [84].

Unfortunately, in the case of tomato allergens the problem is more complex as proteins such as profilin (Sola l 4) or intracellular pathogenesis-related protein (Sola l 4) present high cross-reactivity. For instance, cross-reactivity to tomato was described for up to 28% of latex-allergic patients [55]. Foetisch et al. (2001) [35] showed that 9% of birch-pollen-allergic patients had adverse reactions to tomato. In another study, published by de Martino et al. (1988) [54], 39% of grass-pollen-allergic children were sensitized to tomato. Unfortunately, in the case of tomato profilin the cross-reactivity is mainly caused by the high percentage of structure similarity between profilin and other allergens derived from different fruits, vegetables, or grass pollen. That excludes the use of the above-mentioned tests for anti-CCD IgE antibodies because the CCDs in these particular circumstances are not the reason for the mistakes in widely used immunoassays. The importance of cross-reactivity and the solution to the problem it causes can be one of the most urgent research topics in the upcoming years, although the occurrence of cross-reactivity may also be helpful. For instance, the high sequence similarity allows the use of already designed IgE antibodies to detect new allergens that were not known previously. 

## 9. Transgenic Tomatoes

Taking into the consideration the chemical and physical diversity between all tomato allergens, it is not easy to find one proper strategy to neutralize their influence on an allergic person, other than to exclude tomatoes from their daily diet. It seems that the tools of molecular biology may bring the solution for this problem in the form of genetically modified (GM) plants. For at least the last twenty years, the progression of the methods and tools in the field of genetics and molecular biology has increased extremely fast, giving us new opportunities. One of them is the development of hypoallergenic food.

One of the first attempts to create transgenic tomatoes was made by Lien et al. in 2006 [86]. This research focused on silencing the genes encoding the profilins (Sola l 1). To reduce the amount of profilin (Sola l 1) in tomato fruits, the RNAi (interference RNA) was used. The results from a Northern blot analysis of isolated RNA from transgenic tomatoes and from an ELISA inhibition assay confirmed that Le et al. (2006) [86] had succeeded. The profilin accumulation in transgenic fruits decreased 10-fold in comparison with the wild type. Although the allergenic reactivity of Sola l 1 was reduced, growth retardation was also observed in Sola l 1-silenced plants. The transgenic plants were smaller and formed fewer fruits. This study has shown that profilin is essential for normal plant development. In their following research, Le et al. (2010) [87] provided the development of hypoallergenic tomatoes by creating transgenic tomatoes, expressing non-allergenic yeast profilin. The researchers used non-plant profilin from yeast (PFY1) to dismiss the retardation growth of the plants. The sequence of yeast profilin shares only 32.6% of the amino acid sequence identity with Sola l 1 and has much lower IgE-binding capacity, which minimizes the risk of cross-reactivity. In this research, they proved, by using in vitro and in vivo assays, that PFY1 substitution in tomatoes did not increase the allergenic potential of transgenic tomato fruits. Patients sensitive to Sola l 1 showed no allergic reaction to PFY1. Furthermore, Le et al. (2010) [87] proved that growth retardation and significant deterioration of the phenotype of Lyc e 1-silenced tomato plants could be repaired by overexpression of PFY1.

A similar strategy to that described above was provided for silencing the genes encoding Sola l 3. Simultaneously, in two studies, RNAi was used to limit the amount of nsLTPs in tomatoes. Lien et al. (2006) [88] started with the identification and molecular characterization of the then newly recognized Sola l 3 (previously Lyc e 3). In this study, they followed the ectopic expression of LTP1 and LTP2 sequences (responsible for coding nsLTPs protein) and conducted immunoblotting analysis to verify the IgE reactivity towards selected proteins. The next step of this study was the creation of transgenic tomato plants which were expressing LTPG1- or LTPG2-specific double-stranded RNA interference (dsRNAi). The Northern and Western blotting results confirmed the silencing of Sola l 3, which resulted in the decrease in nsLTP accumulation in the tomato fruits. Subsequently, using the histamine release from sensitized human basophils, the allergenic potential of transgenic fruits was measured. The results of these assays revealed a 10- to 100-fold decrease in histamine release of human basophils when comparing the transgenic fruit extracts with control extracts. Another successful research with the use of RNAi for silencing Sola l 3 was conducted by Lorenz et al. (2006) [89]. This research was conducted in a similar way to the one described above, with two differences. Firstly, in this study the reduction in the allergenicity of the transgenic fruits was additionally tested by a skin prick test (in vivo). Secondly, Lorenz et al. also (2006) [89] investigated the heritability of silencing genes on the next generation of plants. The SPT test brought better results for transgenic plants. The mean skin reactivity for WT (wild type fruits) ranged between 6–12.5 mm, while for transgenic fruits it varied from 2–10 mm in diameter. As a positive control, histamine dihydrochloride was used, giving the diameters from 7–10 mm. The statistical analysis showed the significant differences favorable for transgenic fruits. To analyze the heritability of the transgene effect, 30 seeds of T0 transgenic lines were chosen for cultivation. Ten chosen seedlings (T1) were then selected for growth in the greenhouse and to be used in further analysis. Afterwards, the immunoblotting test was carried out in terms of verifying the effect of silencing Sola l 3 in the next generation of transgenic plants. The Cor a 8-specific rabbit serum was used in this test, and a strong cross-reaction with nsLTPs from WT was discovered. The transgenic plant lines did not show a similar correlation, which confirms the stability of the process of RNAi-mediated gene silencing

Genetically modified (GM) plants may be the answer to the problem of food allergies, as was shown in the described studies. However, it is important to pay attention to the fact that most patients are not monosensitized. Hence, to create hypoallergenic tomatoes it is necessary to simultaneously silence multiple allergens.

## 10. The Impact of Environmental Conditions and Processing Techniques on Tomato Allergens

Undeniably, the existence of allergens may impede the selection of a daily diet for allergic patients. Many studies were conducted in order to develop a processing technique which may degrade allergens or at least limit their amount in processed food. Although, genetic engineering may work for the elimination of some allergens (such as those described above with Sola l or Sola l 3), it is important to remember that most patients are not monosensitized to one allergen. Another important factor associated with GM is the acceptance of consumers. 

An alternative to genetically modified plants can be the selection of conditions for cultivation that reduce the allergenic potential of tomatoes or the development of a method of tomato processing that eliminates allergic proteins, although the latter will not provide the raw tomatoes which will be allergen-free.

### 10.1. Environmental Conditions

Dölle-Bierke et al. (2011) [90] conducted research in which they examined six different tomato cultivars which were previously cultivated in different environmental conditions. The allergenic potential of the cultivated tomatoes was tested by a skin prick test. The tomatoes were grown with different nitrogen forms (nitrogen, ammonium, and nitrate), which were supplied either in deficient or excessive amounts. Other tested conditions were salinity, various light intensities, and the low and high electrical conductivity of the provided nutrient solutions. However, none of tested environmental conditions had an influence on the degree of skin reactivity in tomato-allergic patients. However, it is important to consider that the provided environmental conditions were performed with only one cultivar of tomatoes.

Further extensive research focusing on the method of cultivation was conducted by Słowianek et al. (2016) [91]. A number of chosen allergens (Profilin, LTP, and homolog of Sola l 4) were examined in tomatoes which were cultivated separately in an organic and a conventional system. The research was conducted for three years. Moreover, in this study both cultivation systems were employed with five different cultivars. The obtained results point out that the correlation between the content of allergens in fruits and the cultivar is statistically significant for all examined allergens. However, the profilin was the only allergen which exhibited a correlation between the used method of cultivation and the allergen content. In this case, tomatoes from organic cultivars showed a higher allergenic potential. As the cultivars were grown in foil tunnels on different farms, the authors also considered the fact that apart from the provided similar growth conditions, different stress factors may have influenced the content of allergens.

### 10.2. Food Processing

There are plenty of studies which indicate that there is a relationship between the methods of food processing and the immunoreactivity of chosen allergens. To those processing methods belong boiling, roasting (thermal methods), high-pressure processing, enzyme hydrolysis, pulsed electric field, fermentation, cold plasma, or ultrasounds (non-thermal methods). The most well-known and probably the most broadly used are the thermal methods and enzymatic hydrolysis. Nevertheless, those two methods do not work for the major tomato allergens, profilins and LTPs. As was mentioned before, tomato profilins are resistant to heat or papain treatment [92]. The same goes for LTPs, which in general are resistant to protease digestion, as was proved by Asero et al. (2000) [38]. In this study, the resistance of LTP against pepsin treatment was examined. In fact, plenty of studies confirmed the persistence of LTP allergenicity to thermal treatments [75,93]. Pravettoni et al. (2011) [93] conducted research in which 22 processed tomato products were investigated in terms of their allergenic impact. The obtained results from the group of ten patients reveals that none of the investigated technological processes reduced the IgE binding to tomato LTP in LTP-positive patients [93].

Further research focusing on the tomato processing and its influence on tomato allergenicity was conducted by Primavesi et al. (2011) [94]. The authors investigated the impact of chemical peeling and thermal treatment on the IgE-binding capacity of tomato LTP. Both examined methods did not significantly affect the IgE-binding capacity of lipid transfer protein. The results provided from IgE immunoblotting and the skin prick test revealed that both fresh and chemically peeled tomatoes showed the same allergenic potential. In this research, the thermal stability of LTP was also proven.

To the best of the authors’ knowledge, there was no research conducted focusing on the other methods of food processing (except that mentioned above) in terms of the reduction in tomato allergenicity.

## 11. Current State of the Art

Over the past two decades, tomato allergen research has focused primarily on attempting to identify new, undescribed putative allergens. Unfortunately, progress in this area appears to be quite slow. For instance, as it was mentioned earlier, D’Agostino et al. (2019) [72] detected more than 60 sequences in the tomato genome that probably encode nsLTP, and their study was published in 2019. Through the years, a lot of IgE-binding proteins have been reported in the literature as putative tomato allergens, and valid information about them is available at the Allergome database (http://www.allergome.org/index.php (accessed on 23 March 2022). However, only preliminary data on their allergenicity is currently available [95]. There are also studies concerning the phenomenon of cross-reactivity between different allergens. Mócsai et al. (2017) [95] carried out studies on the identification of the major allergens and evaluated the differences in the IgE-binding properties of these proteins. The result once again pointed out the problem with the detection methods. At the moment, there is a relatively large information pool on the subject of the tomato allergy, but the data are often not sufficiently consistent and do not give a clear picture on the subject.

## 12. Concluding Remarks

Despite many years of research on tomato allergy and specific tomato allergens, our knowledge in this field is still not satisfactory. Every year new information enlarges the available data, and new analytical methods are designed. Still, the immunology-based methods, which are often used in allergen identification, are quite problematic because of the occurrence of cross-reactivity. The phenomenon of cross-reactivity can be considered as a critical point in the usage of immune assays, causing recurring false results. There are other aspects of the studies on tomato allergy that should also be elaborated. One of them is described in the previous section on the influence of cultivation conditions on the allergenic potential of plants. There is still much to discover to fully understand all the processes underlying the occurrence of allergies and their efficient prevention.

## Figures and Tables

**Table 1:** Vitamin and mineral content of tomato [5].

Description	Units	Concentration
Calcium (Ca)	mg/100 g	105.21 ± 22,76
Phosphorus (P)	mg/100 g	300.99 ± 32.12
Iron (Fe)	mg/100 g	4.55 ± 2.18
Sodium (Na)	mg/100 g	70.38 ± 12.20
Potassium (K)	mg/100 g	403.02 ± 254.41
Magnesium (Mg)	mg/100 g	172.58 ± 58.92
Vitamin A	IU/100 g	614.44 ± 248.18
Vitamin E	μg/100 g	15.08 ± 1.06
Thiamine	mg/100 g	0.66 ± 0.44
Riboflavin (mg)	mg/100 g	0.48 ± 0.34
Niacin (mg)	mg/100 g	9.68 ± 0.00
Ascorbic acid (mg)	mg/100 g	36.16 ± 29.64

**Table 2:** The top ten tomato-producing countries. Annual data 2014–2016 [6].

Country	Total Production (×10^6^ t)	Tomatoes for Processing	
		(×10^6^ t)	(% Total)
China	55.72	5.60	11
India	18.74	0.13	<1
USA	14.51	13.40	95
Turkey	11.85	2.70–3.9	25–30
Egypt	8.29	0.25	3
Iran	5.97	1.35	23
Italy	5.62	5.40	96
Brazil	4.30	1.30	33
EU	17.90	60	40
World total	171	41	24

**Table 3:** Tomato allergens, all officially listed by The International Union of Immunological Societies *.

Plant Species	Allergen	Biochemical Name	MW (SDS-PAGE)
*Solanum lycopersicum* (*Lycopersicon esculentum*)—Tomato	Sola l 1	Profilin	14 kDa
	Sola l 2	Beta-fructofuranosidase	50 kDa
	Sola l 3	Non-specific lipid transfer protein type 1 (nsLTP1)	9 kDa
	Sola l 4	Pathogenesis-related protein, PR-10, Bet v 1 family member, TSI-1	20 kDa
	Sola l 5	Cyclophilin	19 kDa
	Sola l 6	Non-specific lipid transfer protein type 2 (nsLTP2)	7 kDa
	Sola l 7	nsLTP type 1	12.5 kDa (SDS PAGE reducing)

* Data available on http://www.allergen.org/index.php (accessed on 23 March 2022).

**Table 4:** Chosen allergens belonging to the Profilin Family *.

Plant Species		Allergen Name	UNIPROT	Id (%)
Tomato	*Solanum lycopersicum*	Sola l 1	Q93YG7	100
Bell Pepper	*Capsicum annuum*	Cap a 2	Q93YI9	91.6
Para rubber tree (Latex)	*Hevea brasiliensis*	Hev b 8	O65812	84.7
Pineapple	*Ananas comosus*	Ana c 1	Q94JN2	80.6
Apple	*Malus domestica*	Mal d 4	Q9XF42	78.6
Carrot	*Daucus carota*	Dau c 4	Q8SAE6	77.6
Celery	*Apium graveolens*	Api g 4	Q9XF37	76.9
*Betula pendula*	*Betula erucosa*	Bet v 2	P25816	74.4

* Sequence identity to Sola l 1.

**Table 5:** Glycan structures identified in natural protein of Sol l 2 derived from tomato [37].

Sugar Moiety	Mol-% in Sol l 2
MUXF3 (Mana1–6(Xylb1–2)Man b1–4GlcNAcb1–4(Fuca1–3)GlcNAc)	5.3
MMX (Mana1–6(Mana1–3)(Xylb1–2)Manb1–4GlcNAcb1–4GlcNAc)	8.2
MMXF3 (Mana1–6(Mana1–3)(Xylb1–2)Man b1– 4GlcNAcb1–4(Fuca1–3)GlcNAc)	83.6
GnMXF3 (GlcNAcb1–2Mana1–6(GlcNAcb1–2Mana1–3)(Xylb1–2)Manb1–4GlcNAcb1–4(Fuca1–3)GlcNAc)	2.3
GnGnMXF3 (GlcNAcb1–2Mana1–6(Mana1–3)(Xylb1–2)Manb1–4GlcNAcb1–4(Fuca1–3)GlcNAc)	0.6

**Table 6:** Members of the nsLTP.

Plant Species	Bionomial Name	nsLTP	UNIPROT
Tomato	*Solanum lycopersicum*	Sol l 3	P93224
Peach	*Prunus persica*	Pru p 3	P81402/Q9LED1
Apple	*Malus domestica*	Mal d 3	Q5J026
Apricot	*Prunus armeniaca*	Pru ar 3	P81651
Plum	*Prunus domestica*	Pru d 3	P82534
Cherry	*Prunus avium*	Pru av 3	Q9M5X8
Strawberry	*Fragaria ananassa*	Fru a 3	Q8VX12/Q4PLT9/Q4PLU0/Q4PLT6
Lemon	*Citrus limon*	Cit l 3	P84160
Asparagus	*Asparagus officinalis*	Aspa o 1	-
Lettuce	*Lactuca sativa*	Lec s 1	-
Cabbage	*Brassica oleracea*	Bra o 3	-
Latex	*Hevea brasiliensis*	Hev b 12	Q8RYA8
Parietaria	*Parietaria judaica*	Par j 1	P43217/O04404/Q1JTN5/Q40905
Ragweed	*Ambrosia artemisiifolia*	Amb a 6	O04004
Olive	*Olea europoea*	Ole e 7	P81430
Celery	*Apium graveolens*	Api g 2	E6Y8S8
Peanut	*Arachis hypogaea*	Ara h 9	B6CEX8/B6CG41
Asparagus	*Asparagus officinalis*	Aspa o 1	-
Chestnut	*Castanea sativa*	Cas s 8	-
Mugwort	*Artimisia vulgaris*	Art v 3	P0C088/C4MGG9/C4MGH0/C4MGH1

## References

[1] Peralta I.E., Knapp S., Spooner D.M. (2009). New Species of Wild Tomatoes (*Solanum* Section *Lycopersicon*: Solanaceae) from Northern Peru. Syst. Bot..

[2] Sahlin E., Savage G., Lister C. (2004). Investigation of the Antioxidant Properties of Tomatoes after Processing. J. Food Compos. Anal..

[3] Tilahun S., Park D., Seo M., Jeong C. (2017). Review on Factors Affecting the Quality and Antioxidant Properties of Tomatoes. AFRICAN J. Biotechnol..

[4] Sainju U., Dris R., Singh B. (2003). Mineral Nutrition of Tomato. Food Agric. Environ..

[5] Vashisth A., Nagarajan S. (2009). Effect on Germination and Early Growth Characteristics in Sunflower (*Helianthus annus*) Seeds Exposed to Static Magnetic Field. J. Plant Physiol..

[6] Costa M., Heuvelink E. (2018). The Global Tomato Industry.

[7] Onopiuk A., Półtorak A., Wojtasik-Kalinowska I., Szpicer A., Marcinkowska-Lesiak M., Pogorzelski G., Wierzbicka A. (2019). Impact of the Storage Atmosphere Enriched with Ozone on the Quality of *Lycopersicon esculentum* Tomatoes. J. Food Process. Preserv..

[8] Kucuk O., Sarkar F., Sakr W., Djuric Z., Pollak M., Khachik F., Li Y.W., Banerjee M., Grignon D., Bertram J.S. (2001). Phase II Randomized Clinical Trial of Lycopene Supplementation before Radical Prostatectomy. Cancer Epidemiol. Biomark. Prev..

[9] Nishino H., Murakoshi M., Ii T., Takemura M., Kuchide M., Kanazawa M., Yang Mou X., Wada S., Masuda M., Ohsaka Y. (2002). Carotenoids in Cancer Chemoprevention. Cancer Metastasis Rev..

[10] Riccioni G. (2009). Carotenoids and Cardiovascular Disease. Curr. Atheroscler. Rep..

[11] Hossin I., Talukdar G., Roy N., Shaha R. (2012). Anti-Allergic Compounds from Red Tomato Peel. J. Adv. Lab. Res. Biol..

[12] Capanoglu E., Beekwilder J., Boyacioglu D., De Vos R.C.H., Hall R.D. (2010). The Effect of Industrial Food Processing on Potentially Health-Beneficial Tomato Antioxidants. Crit. Rev. Food Sci. Nutr..

[13] Chen R.Y., Wu J.J., Tsai M.J., Liu M.S. (2000). Effects of Storage and Thermal Treatment on the Antioxidant Activity of Tomato Fruits. Taiwan J. Agric. Chem. Food Sci..

[14] Graziani G., Pernice R., Lanzuise S., Vitaglione P., Anese M., Fogliano V. (2003). Effect of Peeling and Heating on Carotenoid Content and Antioxidant Activity of Tomato and Tomato-Virgin Olive Oil Systems. Eur. Food Res. Technol..

[15] Chang C.-H., Lin H.-Y., Chang C.-Y., Liu Y.-C. (2006). Comparisons on the Antioxidant Properties of Fresh, Freeze-Dried and Hot-Air-Dried Tomatoes. J. Food Eng..

[16] Alda L., Gogoaşă I., Bordean D.-M., Gergen I., Alda S., Moldovan C., Niţă L. (2009). Lycopene Content of Tomatoes and Tomato Products. J. Agroaliment. Process. Technol..

[17] Yamamoto T., Yoshimura M., Yamaguchi F., Kouchi T., Tsuji R., Saito M., Obata A., Kikuchi M. (2004). Anti-Allergic Activity of Naringenin Chalcone from a Tomato Skin Extract. Biosci. Biotechnol. Biochem..

[18] Gates K.W. (2015). Food Allergen Testing: Molecular, Immunochemical, and Chromatographic Techniques, by George Siragakis and Dimosthenis Kizis. J. Aquat. Food Prod. Technol..

[19] Petersen A., Vieths S., Aulepp H., Schlaak M., Becker W.-M. (1996). Ubiquitous Structures Responsible for IgE Cross-Reactivity between Tomato Fruit and Grass Pollen Allergens. J. Allergy Clin. Immunol..

[20] Ortolani C., Ispano M., Pastorello E.A., Ansaloni R., Magri G.C. (1989). Comparison of Results of Skin Prick Tests (with Fresh Foods and Commercial Food Extracts) and RAST in 100 Patients with Oral Allergy Syndrome. J. Allergy Clin. Immunol..

[21] Burney P.G.J., Potts J., Kummeling I., Mills E.N.C., Clausen M., Dubakiene R., Barreales L., Fernandez-Perez C., Fernandez-Rivas M., Le T.-M. (2014). The Prevalence and Distribution of Food Sensitization in European Adults. Allergy.

[22] Ballmer-Weber B., Hoffmann-Sommergruber K. (2011). Molecular Diagnosis of Fruit and Vegetable Allergy. Curr. Opin. Allergy Clin. Immunol..

[23] Geroldinger-Simic M., Zelniker T., Aberer W., Ebner C., Egger C., Greiderer A., Prem N., Lidholm J., Ballmer-Weber B., Vieths S. (2011). Birch Pollen-Related Food Allergy: Clinical Aspects and the Role of Allergen-Specific IgE and IgG(4) Antibodies. J. Allergy Clin. Immunol..

[24] Bencivenni M., Faccini A., Bottesini C., Rao R., Detoraki A., Ridolo E., Marone G., Dall’Aglio P., Dossena A., Marchelli R. (2012). Assessing Allergenicity of Different Tomato Ecotypes by Using Pooled Sera of Allergic Subjects: Identification of the Main Allergens. Eur. Food Res. Technol..

[25] López-Matas M.A., Larramendi C.H., Huertas A.J., Ferrer A., Moya R., Pagán J.A., Navarro L.A., García-Abujeta J.L., Carnés J. (2015). Tomato NsLTP as an “In Vivo” Diagnostic Tool: Sensitization in a Mediterranean Population. J. Investig. Allergol. Clin. Immunol..

[26] Asero R., Antonicelli L., Arena A., Bommarito L., Colombo G., Crivellaro M., De Carli M., Della-Torre E., Della Torre F., Heffler E. (2009). Causes of Food-Induced Anaphylaxis in Italian Adults: A Multi-Centre Study. Int. Arch. Allergy Immunol..

[27] Zacharisen M.C., Elms N.P., Kurup V.P. (2002). Severe Tomato Allergy (*Lycopersicon esculentum*). Allergy Asthma Proc..

[28] Worm M., Edenharter G., Ruëff F., Scherer K., Pföhler C., Mahler V., Treudler R., Lang R., Nemat K., Koehli A. (2012). Symptom Profile and Risk Factors of Anaphylaxis in Central Europe. Allergy.

[29] Coombs R., Gell P., Gell P.G.H., Coombs R.R.A. (1963). The Classification of Allergic Reactions Underlying Disease. Clinical Aspects of Immunology.

[30] Żukiewicz-Sobczak W.A., Wróblewska P., Adamczuk P., Kopczyński P. (2013). Review Paper: Causes, symptoms and prevention of food allergy. Adv. Dermatol. Allergol..

[31] Pravettoni V., Primavesi L., Farioli L., Brenna O., Pompei C., Conti A., Scibilia J., Piantanida M., Mascheri A., Pastorello E. (2009). Tomato Allergy: Detection of IgE-Binding Lipid Transfer Proteins in Tomato Derivatives and in Fresh Tomato Peel, Pulp, and Seeds. J. Agric. Food Chem..

[32] Bässler O.Y., Weiss J., Wienkoop S., Lehmann K., Scheler C., Dölle S., Schwarz D., Franken P., George E., Worm M. (2009). Evidence for Novel Tomato Seed Allergens: IgE-Reactive Legumin and Vicilin Proteins Identified by Multidimensional Protein Fractionation−Mass Spectrometry and in Silico Epitope Modeling. J. Proteome Res..

[33] Bleumink E., Berrens L., Young E. (1966). Studies on the Atopic Allergen in Ripe Tomato Fruits. I. Isolation and Identification of the Allergen. Int. Arch. Allergy Appl. Immunol..

[34] Bleumink E. (1970). Food Allergy The Chemical Nature of the Substances Eliciting Symptoms. World Rev. Nutr. Diet..

[35] Foetisch K., Son D., Altmann F., Aulepp H., Conti A., Haustein D., Vieths S. (2001). Tomato (*Lycopersicon esculentum*) Allergens in Pollen-Allergic Patients. Eur. Food Res. Technol..

[36] Kondo Y., Urisu A., Tokuda R. (2001). Identification and Characterization of the Allergens in the Tomato Fruit by Immunoblotting. Int. Arch. Allergy Immunol..

[37] Westphal S., Kolarich D., Foetisch K., Lauer I., Altmann F., Conti A., Crespo J., Rodríguez J., Enrique E., Vieths S. (2003). Molecular Characterization and Allergenic Activity of Lyc e 2 (β-Fructofuranosidase), a Glycosylated Allergen of Tomato. Eur. J. Biochem..

[38] Asero R., Mistrello G., Roncarolo D., de Vries S.C., Gautier M.-F., Ciurana C.L.F., Verbeek E., Mohammadi T., Knul-Brettlova V., Akkerdaas J.H. (2000). Lipid Transfer Protein: A Pan-Allergen in Plant-Derived Foods That Is Highly Resistant to Pepsin Digestion. Int. Arch. Allergy Immunol..

[39] Westphal S., Kempf W., Foetisch K., Retzek M., Vieths S., Scheurer S. (2004). Tomato Profilin Lyc e 1: IgE Cross-Reactivity and Allergenic Potency. Allergy.

[40] Asero R., Mistrello G., Roncarolo D., Amato S., Arcidiacono R., Fortunato D. (2008). Detection of a Novel Allergen in Raw Tomato. J. Investig. Allergol. Clin. Immunol..

[41] Palomares O., Villalba M., Quiralte J., Polo F., Rodriguez R. (2005). 1,3-β-Glucanases as Candidates in Latex-Pollen-Vegetable Food Cross-Reactivity. Clin. Exp. Allergy.

[42] López M.A., Hernando de Larramendi C., Ferrer A., Huertas A., Pagán J., Garcia Abujeta J., Bartra J., Andreu C., Lavín J., Carnés J. (2011). Identification and Quantification of Tomato Allergens: In Vitro Characterization of Six Different Varieties. Ann. Allergy. Asthma Immunol..

[43] Diaz-Perales A., Collada C., Blanco C., Sanchez-Monge R., Carrillo T., Aragoncillo C., Salcedo G. (1999). Cross-Reactions in the Latex-Fruit Syndrome: A Relevant Role of Chitinases but Not of Complex Asparagine-Linked Glycans. J. Allergy Clin. Immunol..

[44] Krishnan K., Moens P.D.J. (2009). Structure and Functions of Profilins. Biophys. Rev..

[45] Carlsson L., Nyström L.-E., Sundkvist I., Markey F., Lindberg U. (1977). Actin Polymerizability Is Influenced by Profilin, a Low Molecular Weight Protein in Non-Muscle Cells. J. Mol. Biol..

[46] Rodríguez Del Río P., Díaz-Perales A., Sánchez-García S., Escudero C., Ibáñez M.D., Méndez-Brea P., Barber D. (2018). Profilin, a Change in the Paradigm. J. Investig. Allergol. Clin. Immunol..

[47] Darnowski D.W., Valenta R., Parthasarathy M.V. (1996). Identification and Distribution of Profilin in Tomato (*Lycopersicon esculentum* Mill.). Planta.

[48] Zeleny R., Altmann F., Praznik W. (1999). Structural Characterization of the N-Linked Oligosaccharides from Tomato Fruit. Phytochemistry.

[49] Priem B., Gitti R., Bush C.A., Gross K.C. (1993). Structure of Ten Free N-Glycans in Ripening Tomato Fruit. Arabinose Is a Constituent of a Plant N-Glycan. Plant Physiol..

[50] Valenta R., Duchene M., Ebner C., Valent P., Sillaber C., Deviller P., Ferreira F., Focke-Tejkl M., Edelmann H., Kraft D. (1992). Profilins Constitute a Novel Family of Functional Plant Pan-Allergens. J. Exp. Med..

[51] Vieths S., Scheurer S., Ballmer-Weber B. (2002). Current Understanding of Cross-Reactivity of Food Allergens and Pollen. Ann. N. Y. Acad. Sci..

[52] Valenta R., Duchêne M., Pettenburger K., Sillaber C., Valent P., Bettelheim P., Breitenbach M., Rumpold H., Kraft D., Scheiner O. (1991). Identification of Profilin as a Novel Pollen Allergen; IgE Autoreactivity in Sensitized Individuals. Science.

[53] Willerroider M., Fuchs H.C., Ballmer-Weber B., Focke-Tejkl M., Susani M., Thalhamer J., Ferreira F., Wüthrich B., Scheiner O., Breiteneder H. (2003). Cloning and Molecular and Immunological Characterisation of Two New Food Allergens, Cap a 2 and Lyc e 1, Profilins from Bell Pepper (*Capsicum annuum*) and Tomato (*Lycopersicon esculentum*). Int. Arch. Allergy Immunol..

[54] de Martino M., Novembre E., Cozza G., de Marco A., Bonazza P., Vierucci A. (1988). Sensitivity to Tomato and Peanut Allergens in Children Monosensitized to Grass Pollen. Allergy.

[55] Beezhold D.H., Sussman G.L., Liss G.M., Chang N.S. (1996). Latex Allergy Can Induce Clinical Reactions to Specific Foods. Clin. Exp. Allergy.

[56] van Ree R., Voitenko V., van Leeuwen W.A., Aalberse R.C. (1992). Profilin Is a Cross-Reactive Allergen in Pollen and Vegetable Foods. Int. Arch. Allergy Immunol..

[57] Yu L.X., Parthasarathy M.V. (2014). Molecular and Cellular Characterization of the Tomato Pollen Profilin, *LePro1*. PLoS ONE.

[58] Ebner C., Jensen-Jarolim E., Leitner A., Breiteneder H. (1998). Characterization of Allergens in Plant-Derived Spices: Apiaceae Spices, Pepper (Piperaceae), and Paprika (Bell Peppers, Solanaceae). Allergy.

[59] Sturm A., Chrispeels M. (1990). cDNA Cloning of Carrot Extracellular B-Fructosidase and Its Expression in Response to Wounding and Bacterial Infection. Plant Cell.

[60] Yelle S., Chetelat R.T., Dorais M., DeVerna J.W., Bennett A.B. (1991). Sink Metabolism in Tomato Fruit 1: IV. Genetic and Biochemical Analysis of Sucrose Accumulation. Plant Physiol..

[61] Sturm A. (1991). Heterogeneity of the Complex N-Linked Oligosaccharides at Specific Glycosylation Sites of Two Secreted Carrot Glycoproteins. Eur. J. Biochem..

[62] Asero R., Mistrello G., Roncarolo D., Amato S., Caldironi G., Barocci F., Ree R. (2002). Immunological Cross-Reactivity between Lipid Transfer Proteins from Botanically Unrelated Plant-Derived Foods: A Clinical Study. Allergy.

[63] Sánchez-Monge R., Lombardero M., García-Sellés F.J., Barber D., Salcedo G. (1999). Lipid-Transfer Proteins Are Relevant Allergens in Fruit Allergy. J. Allergy Clin. Immunol..

[64] Salcedo G., Díaz-Perales A., Sánchez-Monge R. (1999). Fruit Allergy: Plant Defence Proteins as Novel Potential Panallergens. Clin. Exp. Allergy J. Br. Soc. Allergy Clin. Immunol..

[65] Boutrot F., Guirao A.-L., Alary R., Joudrier P., Gautier M.-F. (2005). Wheat Non-Specific Lipid Transfer Protein Genes Display a Complex Pattern of Expression in Developing Seeds. Biochim. Biophys. Acta.

[66] Douliez J.-P., Michon T., Khalil E., Marion D. (2000). Structure, Biological and Technological Functions of Lipid Transfer Proteins and Indolines, the Major Lipid Binding Proteins from Cereal Kernels. J. Cereal Sci..

[67] José-Estanyol M., Gomis-Rüth F.X., Puigdomènech P. (2004). The Eight-Cysteine Motif, a Versatile Structure in Plant Proteins. Plant Physiol. Biochem..

[68] Yeats T.H., Rose J.K.C. (2008). The Biochemistry and Biology of Extracellular Plant Lipid-Transfer Proteins (LTPs). Protein Sci..

[69] Liu F., Zhang X., Lu C., Zeng X., Li Y., Fu D., Wu G. (2015). Non-Specific Lipid Transfer Proteins in Plants: Presenting New Advances and an Integrated Functional Analysis. J. Exp. Bot..

[70] Liu W., Huang D., Liu K., Hu S., Yu J., Gao G., Song S. (2010). Discovery, Identification and Comparative Analysis of Non-Specific Lipid Transfer Protein (NsLtp) Family in Solanaceae. Genom. Proteom. Bioinform..

[71] Li F., Fan K., Ma F., Yue E., Bibi N., Wang M., Shen H., Hasan M.M.-U., Wang X. (2016). Genomic Identification and Comparative Expansion Analysis of the Non-Specific Lipid Transfer Protein Gene Family in Gossypium. Sci. Rep..

[72] D’Agostino N., Buonanno M., Ayoub J., Barone A., Monti S., Rigano M. (2019). Identification of Non-Specific Lipid Transfer Protein Gene Family Members in *Solanum lycopersicum* and Insights into the Features of Sola l 3 Protein. Sci. Rep..

[73] Edstam M.M., Viitanen L., Salminen T.A., Edqvist J. (2011). Evolutionary History of the Non-Specific Lipid Transfer Proteins. Mol. Plant.

[74] Van Loon L.C., Van Strien E.A. (1999). The Families of Pathogenesis-Related Proteins, Their Activities, and Comparative Analysis of PR-1 Type Proteins. Physiol. Mol. Plant Pathol..

[75] Asero R., Piantanida M., Pinter E., Pravettoni V. (2017). The Clinical Relevance of Lipid Transfer Protein. Clin. Exp. Allergy.

[76] Matricardi P.M., Kleine-Tebbe J., Hoffmann H.J., Valenta R., Hilger C., Hofmaier S., Aalberse R.C., Agache I., Asero R., Ballmer-Weber B. (2016). EAACI Molecular Allergology User’s Guide. Pediatr. Allergy Immunol. Off. Publ. Eur. Soc. Pediatr. Allergy Immunol..

[77] Salcedo G., Sánchez-Monge R., Barber D., Diaz-Perales A. (2007). Plant Non-Specific Lipid Transfer Proteins: An Interface between Plant Defence and Human Allergy. Biochim. Biophys. Acta.

[78] Volpicella M., Leoni C., Fanizza I., Rinalducci S., Placido A., Ceci L. (2015). Expression and Characterization of a New Isoform of the 9 KDa Allergenic Lipid Transfer Protein from Tomato (Variety San Marzano). Plant Physiol. Biochem..

[79] Martín-Pedraza L., Gonzalez Visiedo M., Gomez F., Blanca-Gómez N., Garrido-Arandia M., Rodriguez R., Torres M., Blanca M., Villalba M., Mayorga C. (2016). Two Non-Specific Lipid Transfer Proteins (NsLTP) from Tomato Seeds Are Associated to Severe Symptoms of Tomato-Allergic Patients. Mol. Nutr. Food Res..

[80] Giangrieco I., Alessandri C., Rafaiani C., Santoro M., Zuzzi S., Tuppo L., Tamburrini M., D’Avino R., Ciardiello M., Mari A. (2015). Structural Features, IgE Binding and Preliminary Clinical Findings of the 7 KDa Lipid Transfer Protein from Tomato Seeds. Mol. Immunol..

[81] Sinha M., Singh R.P., Kushwaha G.S., Iqbal N., Singh A., Kaushik S., Kaur P., Sharma S., Singh T.P. (2014). Current Overview of Allergens of Plant Pathogenesis Related Protein Families. Sci. World J..

[82] Wangorsch A., Jamin A., Foetisch K., Malczyk A., Reuter A., Vierecke S., Schülke S., Bartel D., Mahler V., Lidholm J. (2015). Identification of Sola l 4 as Bet v 1 Homologous Pathogenesis Related-10 Allergen in Tomato Fruits. Mol. Nutr. Food Res..

[83] Kurze E., Lo Scalzo R., Campanelli G., Schwab W. (2018). Effect of Tomato Variety, Cultivation, Climate and Processing on Sola l 4, an Allergen from *Solanum lycopersicum*. PLoS ONE.

[84] Yokoi H., Yoshitake H., Mstsumoto Y., Kawada M., Takato Y., Shinagawa K., Sakurai H., Saito K. (2017). Involvement of Cross-Reactive Carbohydrate Determinants-Specific IgE in Pollen Allergy Testing. Asia Pac. Allergy.

[85] Altmann F. (2007). The Role of Protein Glycosylation in Allergy. Int. Arch. Allergy Immunol..

[86] Le L.Q., Mahler V., Lorenz Y., Scheurer S., Biemelt S., Vieths S., Sonnewald U. (2006). Reduced Allergenicity of Tomato Fruits Harvested from Lyc e 1-Silenced Transgenic Tomato Plants. J. Allergy Clin. Immunol..

[87] Le L.Q., Mahler V., Scheurer S., Foetisch K., Braun Y., Weigand D., Enrique E., Lidholm J., Paulus K.E., Sonnewald S. (2010). Yeast Profilin Complements Profilin Deficiency in Transgenic Tomato Fruits and Allows Development of Hypoallergenic Tomato Fruits. FASEB J. Off. Publ. Fed. Am. Soc. Exp. Biol..

[88] Lien L., Lorenz Y., Scheurer S., Fötisch K., Enrique E., Bartra J., Biemelt S., Vieths S., Sonnewald U. (2006). Design of Tomato Fruits with Reduced Allergenicity by DsRNAi-Mediated Inhibition of Ns-LTP (Lyc e 3) Expression. Plant Biotechnol. J..

[89] Lorenz Y., Enrique E., Lequynh L., Fötisch K., Retzek M., Biemelt S., Sonnewald U., Vieths S., Scheurer S. (2006). Skin Prick Tests Reveal Stable and Heritable Reduction of Allergenic Potency of Gene-Silenced Tomato Fruits. J. Allergy Clin. Immunol..

[90] Dölle-Bierke S., Schwarz D., Lehmann K., Weckwerth W., George E., Worm M., Franken P. (2011). Tomato Allergy: Impact of Genotype and Environmental Factors on the Biological Response. J. Sci. Food Agric..

[91] Słowianek M., Skorupa M., Hallmann E., Rembialkowska E., Leszczynska J. (2016). Allergenic Potential of Tomatoes Cultivated in Organic and Conventional Systems. Plant Foods Hum. Nutr..

[92] Kiyota K., Yoshimitsu M., Satsuki-Murakami T., Akutsu K., Kajimura K., Yamano T. (2017). Detection of the Tomato Allergen Sola l 1 and Evaluation of Its Reactivity after Heat and Papain Treatment. Food Agric. Immunol..

[93] Pravettoni V., Primavesi L., Piantanida M., Brenna O.V., Farioli L., Scibilia J., Mascheri A., Pastorello E.A. (2011). Tmoato Industrial Derivatives: Mallardo Reaction and Residual Allergenicity. Clin. Transl. Allergy.

[94] Primavesi L., Pravettoni V., Brenna O.V., Farioli L., Pastorello E.A., Pompei C. (2011). Influence of Technological Processing on the Allergenicity of Tomato Products. Eur. Food Res. Technol..

[95] Mócsai R., Maczó A., Grünwald-Gruber C., Majer-Baranyi K., Adányi N., Milotay P., Czelecz J., Tömösközi-Farkas R. (2017). Investigation of Allergen Proteins in Five Tomato Cultivars. Acta Aliment..

